# Annual Meeting of the New York State Medical Society

**Published:** 1859-03

**Authors:** 


*Annual Meeting of the New York State Medical Society.*—The fifty-second annual meeting of the New York State Medical Society convened in the Common Council Chamber, City Hall, yesterday morning at eleven o'clock.

The society was called to order by Dr. Thomas C. Brinsmade, of Troy, the President. He addressed the members at considerable length, thanking them for the honor conferred on him, in selecting him to preside over their deliberations. He congratulated the society upon its success, and the influence possessed by it, and referred to the value of its transactions as comparing favorably with the transactions of the London Medical Society. Believing that a proper interest was not manifested by the physicians of the State in medical societies, the president addressed circulars to the societies of every county in the State, and received answers from twenty-eight counties. In these counties, little more than one-half of the regular physicians were members of the societies, and not more than one-third of these attended meetings. He dwelt upon this apathy, and called the attention of the society to it, in the hope that it might be remedied.

He recommends an interchange of transactions with other State societies. He approves of the institution of a second degree in the medical profession, and suggested that the first degree be styled Doctor of Medicine, and the second degree, Bachelor of Medicine. He alluded to the necessary steps to be taken to secure these degrees, and commented upon the justice and necessity of this course, believing that it would greatly elevate the profession. There are five medical journals published in the State, and three republished. The medical profession of this State now occupies a higher position than at any other time. He recommends that a suitable delegation be sent to the great medical meeting at Louisville.

On motion of Dr. Alden March, a vote of thanks to the president for his inaugural address was adopted, and a copy requested for publication.

The following committees were appointed by the president:

*On Credentials*—Drs. B. T. Barker, Alexander Thompson, S. B. Willard.

*On Nominations—First District*—Dr. —— Foster; second do., Dr. J. H. Parker; third do., Dr. B. P. Staats; fourth do., Dr. H. Corliss; fifth do., Dr. N. H. Dering; sixth do., Dr. F. Hyde; seventh do., Dr. H. Jewett; eighth do., Dr. F. H. Hamilton.

Dr. A. H. Hoff moved the appointment of a committee to invite the governor and state officers, and the medical members of the legislature, to take seats with the society during the session. Adopted, and Drs. Hoff, Taylor, and Sprague were appointed such committee.

Dr. Sprague moved that so much of the president's address as relates to the republication of important papers in the earlier publications of the society, now mainly out of circulation, be referred to a select committee of three. Adopted, and Drs. Sprague, S. D. Willard, and Thorn, were appointed such committee.

A communication was received from Dr. Thomas McCall, of Utica, regretting his inability to be present with the society, and enclosing a paper entitled "The Commandment of Knowledge, in relation to Medical Doctrines and Methods."

Dr. Parker, of Dutchess county, read a paper on "The Treatment of Varicose Ulcers," which was referred to the publishing committee.

The committee on credentials presented their report, which was accepted.

Dr. Vanderpoel, of Albany, moved that the resolution adopted at the last meeting, appointing a committee to make arrangements for a dinner, be reconsidered, and the resolution laid upon the table. Adopted.

Dr. George Cook, president of the Canandaigua Lunatic Asylum, was made an honorary member of the society.

Dr. William Taylor, of Manlius, moved the appointment of a committee to consider what action, on the part of the society, can be taken, best calculated to insure a more general vaccination throughout the State. Adopted.

The committee to invite the governor and others, to take seats with the society, reported that they had discharged that duty. Report accepted, and committee discharged.

Dr. March invited the members of the society to visit the hospital at one o'clock, to witness the operation of amputation, the patient being Montgomery Bull, whose arm had been terribly lacerated by machinery. The invitation was accepted, and the members proceeded to the hospital, where the operation was performed by Dr. J. H. Armsby, of Albany, the arm being amputated at the shoulder.

The society then took a recess untill three o'clock.

### AFTERNOON SESSION.

The society reconvened at three o'clock.

Dr. Sprague moved the appointment of a committee of three, to request of the Assembly the use of their chamber, in which the annual address may be delivered; and, also, to invite the governor, and the members of the legislature to be present. Adopted.

Drs. J. S. Sprague, B. P. Staats, and Ball, were appointed said committee.

Drs. W. C. Rogers, of Green Island, Swinburne and Moore, of Albany, C. R. Agnew, of New York, and C. R. Millington, of Herkimer, were invited to take seats with the members of the society.

The Chair announced the following committee on Dr. Taylor's resolution, adopted at the morning session: Drs. Wm. Taylor, Blatchford, and Alden March.

A communication on the subject of "Partial Dislocation, Consecutive and Muscular Affections of the Shoulder Joint," by Alfred Mercer, M. D., as read before the Onondaga County Medical Society, was presented, and referred to the publishing committee.

Dr. March presented a sketch of the life of the late Dr. James A. Billings, as read before the Genesee Medical Society, which was referred to the publishing committee.

The censors of the southern district reported that they examined, June 29, 1858, Carl August Ludwig Baur, and finding him qualfied, recommended him to the President for a diploma. Report accepted.

A communication was read from the agent of Messrs. Garmer, Lamoureux & Co., which accompanied specimens of granules and drages, or sugar-coated pills.

A communication from Tilden & Co. was also read, accompanying which, were a variety of medical preparations. They have also forwarded to Dr. Howard Townsend a large numer of specimens for the cabinet of the Albany Medical College.

Dr. John Swinburne, of Albany, read a very interesting paper on "The Treatment of Fractures of the Femur," which was referred to the publishing committee.

Dr. Parker moved that the Pharmaceutical preparations presented to the society be referred to a committee of three, to report at the next meeting.

A brief discussion ensued, in which the impropriety of the society endorsing any particular medicine, was urged by different members.

Other members urged that the society had already adopted a resolution endorsing certain preparations, the efficacy of which were very justly doubted, and the object of appointing the committee was to investigate the subject.

The resolution was adopted by a vote of twenty-four to twenty-one, and Drs. E. H. Parker, Howard, Townsend, and Saunders, were appointed the committee.

A resolution of thanks to Tilden & Co., and Garmer, Lamoureux & Co., for their specimens, was adopted.

Dr. Shove, of Westchester county, read a paper on "Congenial Fissure of the Soft Palate," which was referred to the publishing committee.

Dr. Cook, of Canandaigua, by invitation of the society, made a statement to the society of the establishment and progress of the "Brigham Hall" Lunatic Asylum, at Canandaigua. An application has been made to the legislature to incorporate the institution.

Dr. Alden March, of Albany, read a paper on "The Displacement of the Heart." Referred to the publishing committee.

Dr. Saunders, of Madison, read a paper on "Cerebro-Spinal Meningitis," in Madison county. Referred to the publishing committee.

Dr. Gray, of the State Lunatic Asylum, and Dr. Bailey, of Albany, were invited to take seats with the society.

Dr. Hudson, of New York, exhibited to the Society an artificial leg, explaining its construction, formation, &c.

Dr. S. D. Willard, from the committee appointed at the last meeting "to petition the legislature, asking them to amend the statute, that the State Medical Society may elect permanent members, by senatorial districts, now or hereafter established," reported that the interests of the society would not be promoted by a change in the present statute. The committee believe that the interests of the society might be enhanced by increasing the number of its honorary members residing without this State, and they recommend a change of the by-laws.

The report was adopted.

The society then adjourned until Wednesday morning at ten o'clock.

SECOND DAY.

The society reconvened at ten o'clock yesterday morning, when the minutes were read and approved.

Drs. J. H. Reynolds, of Saratoga, F. M. Hopkins, of Essex county, M. R.

Peck, of Glens Falls, B. F. Ethridge, of Herkimer, Thomas J. Wheeler, of Cattaraugus county, and James Wynn, of New York, were admitted as honorary members to take seats with the society.

Dr. B. F. Barker moved to amend the by-laws, so that the society may elect six honorary members annually, instead of two. Adopted.

A communication was received from the Herkimer County Medical Society, covering a "Dissertation upon the Influence of Vegetation upon Animal Life and Health." Referred to publishing committee.

An address, read before the Sullivan County Medical Society, by John D. Watkins, M. D., on "Pneumonia, Biliosa and Typhoid," was presented, and referred to the publishing committee.

A communication was received from the Albany County Medical Society, covering a paper, read before the Society, by Dr. S. H. Freeman, being a "biographical sketch of the late Hon. Samuel Dickson, M. D." Referred to publishing committee.

An invitation from Gov. Morgan, to visit the executive mansion in the evening, was received and accepted.

Dr. S. D. Willard, secretary, reported that he had exchanged transactions with the State medical societies of Connecticut, New Hampshire and California. Exchanges had also been made with thirty-five foreign societies, and, through the regents of the University, had received several communications in return. He had also received letters from Dr. M. S. Perry, of Boston, Mass., and Dr. Henry S. Dickson, of Charleston, S. C., acknowledging the receipt of honorary diplomas. The secretary also stated that, on looking over the papers of the society, he had found a number of volumes of the transactions of the society from 1832 to 1837, which he had caused to be bound.

A motion was made and adopted, that each member, wishing copies of the volume, be charged seventy-five cents each.

Dr. Mundy, of Staten Island, moved the appointment of a committee of three, to investigate the facts connected with the Quarantine.

The resolution giving rise to a debate, which promised to be extended, Dr. Snow moved to lay it on the table. which motion was adopted.

Dr. Allabeu moved that the Legislature be petitioned to pass a law authorizing physicians and surgeons, when employed by coroners to make *post-mortem* examinations, in cases coming under their notice, to charge a fee commensurate to the services rendered, to be audited by the Board of Supervisors, and paid by the county in which such services were obtained.

After a brief debate, the resolution was laid upon the table.

Dr. Goodrich presented a report of the removal of a tumor from the upper and posterior surface of the cranium; result fatal; by C. E. Isaacs, M. D., one of the surgeons of the Brooklyn City Hospital. Referred to the publishing committee.

Dr. F. H. Hamilton, of Buffalo, read a paper "On Shortening in Fractures in the neck of the Femur." Referred to publishing committee.

Drs. Joseph Lewi, Babcock, Henry March, Adams, and Fondey, of Albany, and Dr. Cullen, of Brooklyn, were admitted to seats with the society.

Dr. James McNaughton, from the committee to whom was referred the several essays, offered in competition for the prize offered by the society, for the best dissertation on scarlet fever, awarded the prize to Henry A. Carrington, of Hyde Park, New York. Report accepted.

A communication was received from the Westchester County Medical Society, covering "Biographical sketches of the deceased physicians of Westchester county. Referred to publishing committee.

Dr. A. H. Hoff, of Albany, presented "an address on the Registration of Diseases, by W. C. Rogers, M. D.," as read before the Albany County Medical Society. Referred to the publishing committee.

Dr. S. D. Willard, of Albany, read a very interesting paper on "Dipthe-rite," or the sore throat disease, so prevalent in Albany.

Dr. J. V. P. Quackenbush, of Albany, read a report, in accordance with a resolution passed at the last annual meeting, on "Inversion of the Uterus," which elicited a very interesting discussion, after which it was referred to the publishing committee.

Dr. Taylor, from the committee to whom was referred the subject of Vaccination, reported that if Vaccination can by any means become general, the loathsome, disgusting, and often fatal disease, the small pox, would be effectually eradicated from the land. The committee believe, however, an action of the Society would be inadequate to insure such a result. It is believed the small pox is more generally prevalent in this State at the present time, than at any former period since the introduction of Vaccination, and this is owing in a great measure to neglect on the part of the public as to Vaccination, aud perhaps to some extent to the imperfect manner in which Vaccination is performed. The committce reccommend that application be made to the Legislature for the passage of a law which shall authorize and empower the trustees of each of the several school districts in the State, to exclude from the benefits of public instruction, all who have not been vaccinated. A resolution directing the appointment of a committee to obtain the passage of a law by the present Legislature in conformity to the plan above suggested was offered.

The report of the committee was accepted, and the resolution adopted.

Dr. Thompson moved that a special committee be appointed, to which shall be referred so much of the President's address, as relates to the conferring of the second degree. Adopted, and Drs. Howard Townsend, Alexander Thompson, and Thos. W. Blatchford, were appointed such committee.

The committeee reported that the use of the Assembly Chamber had been granted to the Society, for the delivery of the President's address, and that the Governor, and other State officers, and members of the Legislature had been invited to attend.

Recess until 3 o'clock.

AFTERNOON SESSION.

The Society reconvened at 3 o'clock.

Dr. Bly presented to the Society an artificial leg, and explained its mechanism.

Dr. Bacon read a paper on "Facial Paralysis." Referred to the publishing committee.

Dr. Foster, of New York, offered the following:

Whereas, It has ever been the pride and glory of the Medical Profession that its function are not limited to the cure, but extend also to the prevention of disease; and whereas, the causes of disease among crowded populations are to a great extent under control, and susceptible of being avoided or removed by judicious sanitary regulation, as has abundantly demonstrated in many instances where such measures have effected great reduction in the bills of mortality; and whereas the first object of every civilized goverment should be to protect the health and lives of the citizens, therefore,

Resolved, That this Society has seen with great satisfaction the progress which the science of Public Hygiene has made in the good opinion of the public, and looks forward to the time when, under the direction of those skilled in this branch of Medical science, the ratio of mortality may be reduced to a minimum.

Resolved, That this Society warmly approves the action of His Honor, the Mayor of the city of New York, in his efforts to place the control of the Sanitary affairs of that city in hands of Medical men, who alone are competent to exercise it.

Resolved, That the Legislature of the State are called upon by every motive of policy and humanity to sustain and promote all such laudable attempts to improve the health and save the lives of the community by the passage of such laws as may be necessary to give them immediate efficiency.

The resolutions were adopted.

Dr. Howard Townsend, of Albany, read a papar on "Hypophosphites." Referred to the publishing committee.

Dr. John Ball, of Brooklyn, read a paper on a case of "Hydrops Sacci Lachrymalis." Referred to the publishing committee.

Dr. James Lee, of Saratoga Co., presented a report of "The Diseases of the County of Saratoga." Referred to the publishing committee.

Dr. Parker, of New York, made an oral report on "Obstetrics," and requested the members of the Society to furnish him with statistical information.

Dr. S. H. French, of Broome Co., expressed himself gratified with the remarks of Dr. Parker.

Dr. Seth Shove, of Westchester Co., presented a " Biographical Sketch of Dr. Geo. C. French, of Westchester Co., prepared and read before the West chester County Medical Society. Refered to publishing committee.

Dr. Horace Willard, of Albany Co., read a paper on "Rupture of the Cul de Sac of the Colon." Referred to the publishing committee.

The Society then adjourned until 10 o'clock, Thnrsday morning.

### THIRD DAY.

The Society convened at 10 o'clock yesterday morning. Minutes read and approved.

In yesterday's report of the proceedings we omitted to state that Dr. Bissell, of Utica, presented a paper on "Misplacement of the Uterus," which was referred to the publishing committee.

J. S. Sprague, from the Committee on so much of the President Address, as relates to the re-publication of such of the Transactions of the Society as are not in circulation, reported in favor of the republication of the Addresses of the Presidents of the Society, for the first twenty-five years of its existence. Report accepted.

Dr. F. H. Hamilton, presented the following papers: " Statistics of 753 Obstetrical Cases," by Dr. N. C. Husted, of the New York Academy of Medicine; also, " Death Rate in the State of New York, according to the last Census," by Dr. Stephen Smith of the Academy. Referred to the publishing committee.

Dr. Goodrich, of Brooklyn, submitted a paper on "Vital Statistics of the City of Brooklyn." Referred to the publishing committee.

Dr. Wynn, of New York, moved that the County Medical Society be requested to furnish the next Annual Meeting of the State Medical Society with a complete list, so far as the facts can be ascertained, of the number of their members in each year, and of those who have died, together with the ages at which death took place. Adopted.

The name of Dr. Turtelot, of Middle Grove, Saratoga, was yesterday printed Dr. Turblot.

Dr. Wynn, of N. Y. made a very interesting statement to the Society, on the subject of Mortality in the United States, and the Mortality on account of Intemperance.

Dr. Edward Duffy, of Albany, was invited to take a seat with the members of the Society.

Dr. B. P. Staats, of Albany, moved that the publishing committee cause as many as may be practicable, of the Addresses of former Presidents of this Society (which have not already been published,) to be published in the Transactions of this Society. Adopted.

Dr. Bissell, of Utica, moved that the committee on statistics be continued, and that the Legislature be requested to publish the usual number of blanks. Adopted.

Dr. F. H. Hamilton, of Buffalo, moved that in the law enacted in the Legislature of this State, during the session of 1827, permitting both parties to testify in all civil suits, our prrofession, in common perhaps with the public generally, have an important interest; and that we therefore earnestly recommend to the several members of the Senate and Assembly from their respective districts, that they resist all attempts for its repeal; unless, indeed, it is fully proven that such repeal is demanded by the public good, whose interest ought certainly to be considered as paramount to the interest of individuals or classes. Adopted.

Dr. Goodrich moved the appointment of a committee of three to inquire into the subject of Anesthetic agency, in regard to its origin and its first introduction into Medical and Surgical practice in the United States, and that the committee report all facts in the premises, of interest to the Medical profession, and report at the next annual meeting. Adopted, 12 to 14, and Drs. Goodrich, Jones and F. H. Hamilton where appointed such committee.

Dr. B. P. Staats, of Albany, called attention to the fact that at the last meeting of the Society, the physicians of Warren or Essex Counties, were requested to investigate the case of the woman, who, it was alleged lived without eating.

Several gentlemen stated that it had been pretty well demonstrated that the case was an imposition.

Dr. Ferguson, however, expressed an opposite opinion, and gave an account of his visit to the woman in question.

The subject was then dropped.

The committee on Dr. Taylor's Vaccination Report, consists of Drs. Taylor, Blatchford and March.

Dr. Mundy of Staten Island, offered a resolution, that, in the opinion of this Society, a Quarantine establishment, the object of which is to prevent the introduction of foreign pestilential and infectious diseases, should, in order to obtain the object desired, be located in an isolated place; and an institution of this character, situated in the midst of a dense population and near large cities, and where there is constant interchange and communication, between such place and the places which it is designed to protect, fails to answer the purposes for which it was established. Laid on the table.

Dr. Coates of Genessee county, moved that a committee be appointed to report at the next meeting, the best method for securing the appointment of a "Commission of Lunacy" for the State of New York. Adopted; and Drs. Coates, Coventry, Gray and Cook were appointed such committee.

Dr. J. V. P. Quackenbush of Albany, Treasurer, reported the receipts for the year to have been $143.39, and the expenditures $88.49, leaving a balance in the hands of the Treasurer of $54.80. Report accepted and referred to the usual committee: consisting of Doctors Sprague, Barnett and Sanders, to examine the accounts.

Dr. Thompson, of Cayuga, offered a resolution that the committees to whom was referred so much of the President's Address as relates the institution of the Second Degree in the Medical Profession, be authorized to present the subject to the Convention of Professors, to be held at Louisville, Ky., in May next, as it may think proper. Adopted.

Dr. Saunders, of Onondaga, moved the appointment of a committee of three, to take into consideration the propriety of condensing, in such a manner—the forms for the Registration of Medical and Surgical Statistics for the use of County Practitioners—as will ensure a better attention to the subject, and to report at the next annual meeting. Adopted, and Drs. Sanders, Orlin, and Cogswell, appointed such committee.

The committee to examine the Treasurer's report, reported they had discharged that duty and found the same correct.

The Society then proceeded to an election of officers for the ensuing year, Drs. Sanders and Burton acting as tellers.

The following officers were elected:—

President—Prof. B. Fordyce Bakker, New York city.
Vice President—Dr. Daniel T. Jones, Onondaga.
Secretary—Dr. Sylvester D. Willard, Albany.
Treasurer—Dr. John V. P. Quackenbush, Albany.

Permanent Members—Drs. Franklin Tuthill, Horace K. Willard, Seth Shove, Uriah Potter, Henry N. Porter, C. M. Crandall, A. J. Dallas, P. H. Strong, John Ball, M. C. Hasbrouck, James P. Boyd, R. L. Allen, John Putman, Stephen Hegadorn, J. F. Trowbridge, H. M. Conger.

Committee of Publication—S. D. Willard, Howard Townsend, A. H. Hoff.

To be recommended to the Regents of the University for the Honorary Degree of Doctor of Medicine—Drs. Peter P. Staats, of Albany; M. H. Cash of Orange; J. M. Sturdevant, of Oneida; Richard Laming, of Tompkins.

For Honorary Members—Drs. Silas Durkee, of Boston; John M. De La Mater, of Ohio.

Nominated for Honorary Members—Drs. Ernest Hart, London, England; John Jeffries, Boston, Mass; Henry Bronson, New Haven; G. Mendenhall, Ohio; W. Fraser, Montreal; Charles I. Isaacs, U. S. Army,

CENSORS.

1st Dist.—John Ball, Peter Van Buren, John McNulty.
2d " J. S. Sprague, S. H. French, Geo. Barr.
3d " B. B. Staats, T. W. Blatchford, P. McNaughton.
4th " Alexander Thompson. G. W. Burwell, A. Van Dyck.

Delegates to the American Medical Association left to be designated on application to the Secretary.

Dr. Staats offered a resolution, tendering a vote of thanks to the President for the impartial manner in which he had discharged the duties of his office.

A vote of thanks was extended also to the Common Council of the city of Albany for the use of their Chamber.

The President, Dr. Brinsmade, the addressed the Society in a few parting words, as follows;

Gentlemen—In retiring from this Chair, the duties of which I have so imperfectly performed, I should do injustice to my feelings should I not most heartily thank you over and over for the assistance, courtesies and kindness I have received from you all and which will always be remembered with great pleasure. I hope you may all return to your homes and ordinary arduous, but honorable duties, and have pleasant remembrances of this meeting.

The Society then adjourned *sine die.*—*Albany Atlas & Argus.*

---

*Return of Prof. Austin Flint.*—Prof. Flint has now been more than a week on his way home from new Orleans, and will be in Buffalo before this number is issued; according to all our accounts, the winter has been as pleasant as possible, and the advantages of teaching, both clinical and didactic, all that could be desired.

